# Commentary on: An exploration of sensory and movement differences from the perspective of individuals with autism

**DOI:** 10.3389/fnint.2015.00020

**Published:** 2015-03-20

**Authors:** Elizabeth B. Torres

**Affiliations:** Psychology, Computer Science, Cognitive Science, Sensory Motor Integration, Rutgers UniversityNew Brunswick, NJ, USA

**Keywords:** autism, sensorimotor control, sensorimotor integration, self advocates, parents

“An exploration of sensory and movement differences from the perspective of individuals with autism” by Robledo, Donnellan, and Strandt-Conroy, was originally published in Autism: The Movement Perspective as part of a Research Topic in *Frontiers in Integrated Neuroscience* (2012–2014). It gained a special place in the Topic, as in the general field of research in autism, by being one of the few studies to systematically study autism from the perspective of those with that lived experience. This paucity is all the more remarkable as there are at least 100,000 research studies published on the topic of autism in the past few decades (Robertson, 2010). It's hard not to argue that the perspective of autistic people themselves has been given too little attention by the research community although, fortunately, there are a growing number of self-accounts that have helped to shed light on that perspective over the last 25 years adding immensely to our understanding (e.g., Grandin, 1995; Williams, 1998; Mukhopadhyay, 2000).

Nonetheless, this paper by Robledo, Donnellan, and Strandt-Conroy occupies a unique position having called upon multiple rigorous social science research methodologies to study in-depth the presence and potential impact of “movement differences” in autism. They define movement differences as a difference, interference or shift in the efficient, effective utilization, and integration of movement; a disruption in the organization and regulation of perception, action, posture, language, speech, thought, emotion, and/or memory (Leary et al., 1999) which broadens and extends the notions of sensory-motor challenges which have drawn some attention from researchers in recent times (e.g., DSM-5). Using interviews, some in person and some with specialized accommodations for the individual, questionnaires and participant-produced data they present us with revealing and often poignant insights into what has often been defined in social terms as “inappropriate behavior.” So Barbara, who has difficulty processing two senses at once chooses to turn her head to limit the visual input and is blamed for this accommodation. This research is an important step beyond the many thousands of studies in the canon showing what autistic people fail to do, or do wrong and little about how they attempt to accommodate to their movement challenges.

The editors of Frontiers and, in particular, the Special Research Issue Autism: The Movement Perspective, were proud to present the 40 papers in that issue, many on topics which do not generally get wide attention. We are particularly proud of this paper that has earned the opportunity to receive even broader readership in a Tier 2 publication.

## Conflict of interest statement

The author declares that the research was conducted in the absence of any commercial or financial relationships that could be construed as a potential conflict of interest.
